# Cannabis use during pregnancy and its effect on the fetus, newborn and later childhood: A systematic review

**DOI:** 10.18332/ejm/168727

**Published:** 2023-08-04

**Authors:** Dimitra-Fanouria Ainiti, Aikaterini Lykeridou, Christina Nanou, Anna Deltsidou

**Affiliations:** 1 General Clinic Euromedica Mitera Crete, Heraklion, Greece; 2 Department of Midwifery, University of West Attica, Athens, Greece

**Keywords:** pregnancy, cannabis, fetus, newborn, childhood

## Abstract

**INTRODUCTION:**

Cannabis and its derivatives are becoming increasingly popular in women’s preferences during pregnancy in order to relieve nausea. The present study examines cannabis use during pregnancy and its effects on the fetus, newborn and later childhood.

**METHODS:**

All primary studies were searched in the databases: PubMed, Scopus, Medline during the period June 2019 to August 2020. The keywords used were ‘pregnancy’, ‘pregnant women’, ‘cannabis’, ‘marijuana’, ‘fetus’, ‘newborn’, ‘childhood’, and combined with ‘AND’ and ‘OR’ Boolean operators. Inclusion criteria were: pregnant users of cannabis as the study group and pregnant non-users of cannabis as the control group; the articles could be in English or in Greek. The exclusion criteria were: unpublished studies, reviews, presentations at conferences, and animal studies.

**RESULTS:**

From the systematic review of the literature, the study included 13 primary research studies in which it was found that the children of mother-user faced: disorders in the sleep cycle, memory problems, hyperactivity, increased chances of low birth weight, prematurity with lower Apgar score in the 1st and 5th minutes and hospitalization in an NICU, DNA methylation at the position CpG.32, and modifications in the brain, especially in the amygdala. In addition, girls had more aggressive behavior at the age of 18 months, shorter breastfeeding period, and neonatal death.

**CONCLUSIONS:**

The use of cannabis during the gestation period by the mother, aggravates the physical and mental development of the fetus, the newborn and the later childhood.

## INTRODUCTION

One of the main symptoms of pregnancy is nausea, especially during the 1st trimester of pregnancy. To relieve symptoms more and more pregnant women turn to cannabis use. The rate of use depends on the trimester of pregnancy. Α study conducted in California between 2009 and 2016 among women who developed nausea during pregnancy and women who did not experience this symptom, shows that there was an increasing trend in cannabis use. Indicatively, the rate of use in the user group increased from 6.5% to 11.1%, while in the group of women who did not experience nausea in pregnancy this percentage increased from 3.4% in 2009 to 5.8% in 2016^1^. Even more important, however, is the effect of cannabis on the developing fetus.

In 1993, the number of newborns that showed disturbances from cannabis use was 18.52 children per 10000 births. By 2014 this number increased to 93.64 children per 10000 births. In total for 2014, these children amounted to 35507^2^.

For the developing fetus, disorders are expected as cannabis products pass through the placenta. The main effects found were: low birth weight, prematurity (childbirth before the 37th week of gestation), young newborns for gestational age or lower head circumference, and increased chances of hospitalization in neonatal Intensive Care Units (NICUs)^3,4^.

In Australia, a major survey found that women users were more likely to give preterm birth. The result did not change even when the parallel effect of tobacco and alcohol was examined^5^.

The period of postpartum and breastfeeding is equally important for the newborn. Through breast milk, the newborn receives all the necessary elements for the shielding of their system and their proper physical and mental development. Studies have also revealed the negative effects of cannabis use during the postpartum period. Thus, it was found that women who breastfed with a high frequency were 5% more likely to use cannabis, and in a study conducted in Colorado, women-cannabis users aged <30 years were 7.4%, and for those aged ≥30 years it was 4%^6^. About 35.8% of the total reported that their habit existed before pregnancy. The consequences of use do not differ between the period of the first 3 days after childbirth compared to the period following birth^6^.

Finally, the effect of prenatal exposure to cannabis on the childhood of the individual remains remarkable. The children of mothers who consumed large amounts of cannabis in the first trimester of pregnancy, experienced higher rates of anxiety, depression as well as behavioral problems at the age of about 2 years. In addition, at the age of 10 years there were increased rates of depression, as for the education level these children faced problems in learning and concentration. It is worth noting that the appearance of depression at the age of 10 years was strongly associated with crime at the age of 14 years^7,8^.

### Aim

The aim of this systematic review was to study the use of cannabis during pregnancy in order to reveal the possible effects on the fetus, the newborn and the later childhood, through primary studies.

## METHODS

### Inclusion and exclusion criteria of studies

The primary step in carrying out this systematic review was to find the relevant primary studies which would be included in the review. The appropriateness of the studies was defined by the use of specific criteria-standards. In each study the participants had to be classified into 2 groups, the users and the non-users of cannabis. Any study that met the criteria, regardless of the country of origin, was accepted. The exclusion criteria were unpublished studies, reviews, presentations at conferences, animal studies.

### Search strategy of the bibliography

**Figure 1:**
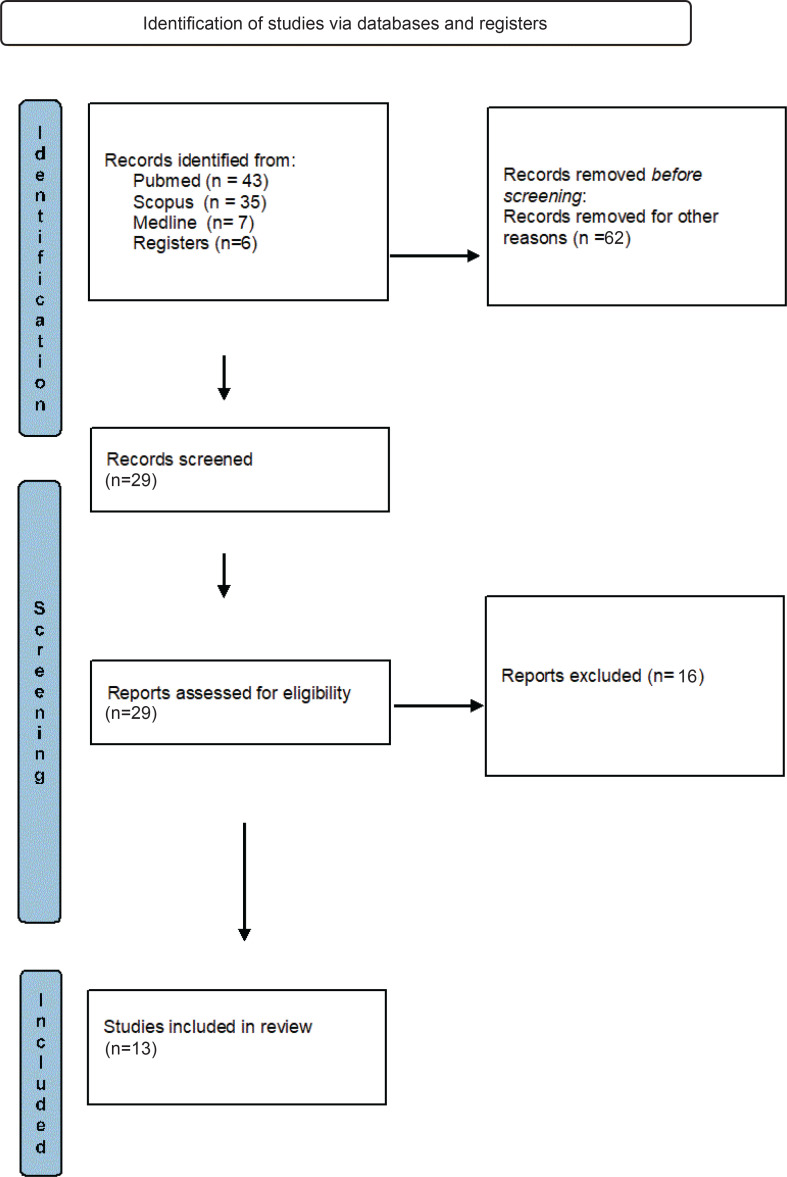
PRISMA flowchart summary of search strategy and study selection

For the purpose of this review, the Scopus, PubMed, Medline databases were searched and the keywords used for the detection of all relevant primary studies were: *pregnancy, pregnant women, cannabis, marijuana, fetal* and *neonatal outcomes* (Table 1). The above terms were combined with the use of the ‘AND’ and ‘OR’ Boolean operators. The search interval took place from June 2019 to August 2020. Searching the abovementioned databases according to the inclusion criteria, a sufficient number of studies was found. In further evaluation, the systematic reviews or metaanalyses were excluded. After the initial screening based on the types of studies, the main guide for evaluation was the title of each project or the summary. The remaining studies were rejected due to the lack of accessibility to the full context. Finally, it was found that some of the studies, after reading the entire text, were not suitable for being included in our review. Thus, the systematic review includes 13 studies that met the criteria and were finally included in the review (Figure 1) and the summary of the results was made with reference to the 13 primary studies, based on the year of publication.

**Table 1:** Search strategy and keywords used to identify studies investigating the relationship between cannabis use in pregnancy and effects on the fetus, newborn and on later childhood

	*Keywords*	*Lookup number*	*Number of studies found from database 1*	*Database search results 2*	*Database search results 3*
Pregnancy	Pregnancy OR pregnant women	#1	1000156	220925	1082
Cannabis use	Cannabis OR marijuana	#2	33768	8905	641
Effects on the fetus, the newborn and on later childhood	Fetal outcomes AND neonatal outcomes	#3	13208	15025	391
Combination	#1 AND #2 AND #3	#4	42	34	7

## RESULTS

### Selection of studies

From the primary search in the three databases, 91 studies, which answered the question: ‘What is the effect on the fetus/newborn from cannabis use during pregnancy’ were recovered. The reading of the titles as well as the summaries of the articles led to the selection of 13 final primary studies which met the criteria required in order to be included in the present systematic review. The rest were rejected because they were either meta-analyses/reviews, or related to an animal study or the title or summary did not cover our subject. The 13 studies that were finally included were evaluated for their methodological quality according to the Ottawa-Newcastle scale. More specifically, the evaluation of the studies is shown in Table 2 and the characteristics of each study are shown in Table 3.

**Table 2:** Methodological quality score of the studies under review (Ottawa-Newcastle scale)

*Study*	*Choice*				*Comparability*		*Result*			*Total*
	*1*	*2*	*3*	*4*	*5*	*6*	*7*	*8*	*9*	
Linn et al. ^9^ (1983)	*	*	-	*	*	-	**	*	-	7
Fried^10^ (1995)	*	*	*	*	-	-	-	*	*	6
Gray et al.^11^ (2010)	*	*	**	*	*	*	-	*	-	8
El Maroun et al.^12^ (2010)	*	*	*	*	*	*	-	*	*	8
Day et al.^8^ (2011)	*	*	*	-	*	*	*	*	*	8
Hayatbakhsh et al.^5^ (2011)	*	*	**	*	*	-	*	*	*	9
Salzwedel et al.^13^ (2015)	*	*	*	-	*	*	*	*	*	8
Leemaqz et al.^14^ (2015)	*	*	*	*	*	*	-	*	-	7
Fransquet et al.^15^ (2016)	*	*	*	*	*	*	-	*	-	7
Crume et al.^16^ (2018)	*	*	*	-	*	*	*	-	*	7
Eiden et al.^8^ (2018)	*	*	*	-	*	*	*	*	*	8
Corsi et al.^17^ (2019)	*	*	**	*	*	*	*	-	-	8
Sturrock et al.^18^ (2019)	*	*	*	*	*	*	-	-	*	7

1) Representative sample of exposure. 2) Selection of exposed. 3) Finding of exposure. 4) The outcome did not exist before the start of the study. 5) Adjustment for education level. 6) Adjustment for additional (secondary) confounding factor. 7) Outcome assessment. 8) Sufficient follow-up time. 9) Non-wear bias.

**Table 3:** Characteristics of studies investigating the relationship between cannabis use in pregnancy and effects on the fetus, the newborn and on later childhood

*Study Year Country*	*Type of study*	*Population (characteristics of participants)*	*Report (measurement)*	*Outcome*	*Results*	*Significance p*	*Adjustments for confounding agents*
Linn et al.^9^ 1983 Boston USA	Retrospective cohort study	12825 women/2529 of them admitted to being cannabis users during pregnancy	Postpartum interview	Newborns had: Lower birth weight Severe malfunctions Lower Apgar score at 1 minute	OR=1.36 for serious malfunctions OR=1.07 for low birth weight	0.001 <0.05	Excluded women who reported drug use
Fried^10^ 1995 Ottawa Ontario Canada	Prospective study	700 women surveyed in pregnancy	Interview during pregnancy in each trimester	Newborns had increased tremor, lower visual acuity At the age of 4 years experienced memory problems	-	-	-
Gray et al.^11^ 2010 USA	Retrospective cohort study	120 pregnant women 12–20 weeks	Interview in each trimester with the completion of a questionnaire Examination of oral smear Collection of meconium from newborns	Newborns had: Lower birth weight Lower head circumference Younger birth age	Low birth weight Shorter week of gestation Shorter head circumference Short body length	<0.051 <0.685 <0.011 <0.156	There had to be a positive test in more than 1 measurement/day of a random sample of meconium. The degree of effect of the fetus with the parallel use of tobacco and cannabis of the mother has a negative outcome.
El Maroun et al.^12^ 2010 Netherlands	Cohort study	5512 children/4077 of them for behavioral problems at the age of 18 months	Questionnaire in the 1st trimester even for use by the partner and examination of a urine sample	Girls had an increased chance of aggressive behavior and concentration problems	OR=1.66 for aggressive behaviors in girls OR=2.75 for concentration matters	p=0.50 for aggressive behavior p=0.01 for concentration problems	Socioeconomic factors, sometimes cannabis use was combined with tobacco use.
Day et al.^7^ 2011 USA	Longitudinal, cohort study	1360 women were selected and their children	Interview in their fourth prenatal month, seventh prenatal month at delivery, 8, and 18 months, and 3, 6,10, 14, 16, and 22 years postpartum. At birth, there were 763 live-born singleton infants.	Depressive symptoms Attention problems Learning and memory problems Delinquency at the age of 14		<0.001 <0.01 <0.01 <0.01	Socioeconomic factors such as household income, home environment etc.
Hayatbakhsh et al.^5^ 2011 Australia	Cohort study	24874 women who gave birth between 2000–2006	Interview in pregnancy	The newborns: Lower birth weight Premature Residual growth Higher chance of going into an Increased Care Unit	ΟR=2.4 OR=1.7 OR=3.1 OR=2.3	<0.001	The age of the mother as well as socioeconomic factors
Leemaqz et al.^13^ 2015 Australia New Zealand Ireland UK	Cohort study	Women who gave birth between November 2004 and February 2011	Asked about cannabis use during the 15th and 20th week of gestation	Prematurity Residual growth	OR=2.31 (95% CI: 1.45–3.55) for prematurity OR=1.37 (95% CI: 0.96–1.92) for newborns with residual growth	<0.001 for prematurity <0.005 for newborns young	Others factors affecting outcome of pregnancy such as PCOS are not included
Salzwedel et al.^14^ 2015 Chatham Orange Durham Alamance North Carolina USA	Prospective study	Newborns were studied in 3 categories	Underwent an rsfMRI test	Abnormal functionality of the amygdala and the islet of the brain of the newborn	-	<0.05–0.001	Cannabis use was associated with education level and the socioeconomic level
Fransquet et al.^15^ 2016 Australia	Cohort study	Study of 1634 women	Questionnaires in pregnancy and after childbirth DNA study in neon after swabbing at 8 weeks of life	DNA methylation in CpG.32 position in newborns exposed to cannabis Methylation in CpG.21.22.23 along with tobacco use	95% CI: 0.11–1.46% 95% CI: 0.02–2.93%	p=0.069 in exclusive use of cannabis p=0.40 with tobacco use	Difficult to separate the effects on the newborn that come exclusively from the use of cannabis.
Crume et al.^16^ 2018 Colorado USA	Cross-sectional study	Women who gave birth from 1/1/2014 to 31/12/2015 in Colorado	Interview by phone and questionnaires, up to 2 months after giving birth	Low birth weight Prematurity Hospitalization in neonatal units Young for birth age newborns	OR=1.8	0.0008 0.2 0.9 0.03	Medical history in pregnancy Education level Nationality Age Socioeconomic level
Eiden et al.^8^ 2018 USA	Prospective study	247 mother-infant dyads	1. Prenatal assessments were conducted once in each trimester of pregnancy 2. A phone interview at 3 years of child age 3. Maternal interviews and infant saliva samples at 2, 9, 16 months, and visits at 2 years	1. Girls in the control group were breastfed longer 2. At 2 years, higher first trimester cannabis use was associated with higher symptoms of anxiety/depression 3. Girls with positive meconium for cannabis had fewer sleep problems		<0.07 <0.05	The sample overall consisted primarily of young, unmarried, lowincome, minority women with low education.
Corsi et al.^17^ 2019 Ontario Canada	Retrospective cohort study	Women >15 years who gave birth to 1 newborn (>20 weeks) in Ontario from 1/4/2012 to 31/12/2017	Asked about cannabis use in routine pregnancy screening	Childbirth before 37 weeks Residual growth Stillbirth Transfer to an Increased Care Unit, lower Apgar score in the 5th minute	95% CI: 5.22–6.54%	<0.001	Many women out of fear concealed the use. There may be risk factors which are not included.
Sturrock et al.^18^ 2019 UK	Cohort study	4465 children born from 1/8/2017 to 31/7/2018	Information about: Habits in pregnancy Socioeconomic level Race The possible hospitalization of newborns in Intensive Care Units	Newborns: Less birth weight Lower head circumference	95% CI: (-0.989 vs -0.587) body weight 95% CI: (-1.33 vs 0.782) head circumference	p=0.028 for birth weight p=0.025 for head circumference	-

### Description of the selected studies

The 13 primary studies included in this review were carried out in several countries around the world. More specifically, 8 took place in the USA (North Carolina, Chatham, Orange County, Colorado, Boston, New York) and Canada and the remaining 5 in Europe (UK, Netherlands, Ireland), New Zealand and Australia. A total of 809355 women and 140689 newborns were included in the 13 studies, and they were approached by various methods. A variety of methods were used: combination questionnaires with interviews^5,9-11^, questionnaires^11-13^ alone, questionnaires with telephone communication^14^ or with rsfMRI in newborns^15^. Generally the majority used questionnaires^11-13,16-18^ while in one case^11^ additional methods were used such as oral fluid intake from mothers, meconium intake from newborns in the first days of life, and blood test in mother. An important bias factor in the studies in which questionnaires were used exclusively, was the honesty of women’s responses^13,17^. Especially in countries where cannabis use was prohibited, the bias was greater in fear of stigmatization. In all studies, the effect of cannabis use during pregnancy on the fetus and the newborn was presented, while in some of them the results of the simultaneous use of two aggravating factors such as cannabis and tobacco were presented^16,18^. The background of the women played an important role in their participation. Women, for example, with a twin pregnancy could not participate as this factor may affect the newborn to an extent that it cannot be clarified whether this effect was due to cannabis or twin pregnancy^9,14,17^. The age of the mother differed from study to study. In some cases, the limit was over 18 years old ^5,11,14^ while in others, women at the age of 15 years could participate^17^.

### The effect of cannabis use on the fetus, the newborn and the later childhood

The treatment of the 13 primary studies found that cannabis use in pregnancy has an aggravating role in the physical and mental development of the fetus and the newborn. Regarding physical development, the newborns of user mothers experienced: tremors, convulsions, disturbances in the sleep cycle, problems of memory-understanding-perception in childhood, and were overactive^10^. Furthermore, cases of preterm birth^5,9,13,14,17^ were recorded, while newborns had a lower birth weight^5,9,11,14,18^ and a lower Apgar Score at 1st minute^9^. In cases where two aggravating factors such as cannabis and tobacco were studied simultaneously, the results were similar^16,18^. In cases where the mother’s history was taken seriously, the consequences of cannabis use were not found to differ^9,13,17^. In fact, a study in Colorado additionally found that mother-user babies breast pumped for a shorter period of time^14^. At the same time, however, in addition to the physical effect, it was found that newborns of cannabis-users were more likely to develop more aggressive behavior at the age of 18 months, especially girls, as well as distraction^12^. This difference between the two sexes is based on the fact that the central nervous system of females develops faster. In addition, at the age of about 2 years, the children of mothers of users showed symptoms of depression and anxiety. An important role in the appearance and severity of these symptoms is played by the rate of cannabis use in the 1st trimester of pregnancy. The more cannabis a mother consumed in the 1st trimester of pregnancy, the more frequent was the occurrence of disturbances at 24 and 36 months of life. Learning difficulties of children at the age of 10 years combined with the appearance of delinquent behavior at the age of 14 years. Children who had faced learning difficulties at 10 years of age had more chances of such kind of behavior occurring^7,8^. As far as DNA is concerned, methylation was detected at CpG 32, while if the mother used an additional aggravating agent at the same time, methylation was also detected at CpG 21.22.23^16^. Finally, with the help of rsfMRI there have been recorded cases of modification in brain function, especially at the site of the amygdala^15^.

## DISCUSSION

From all the primary studies included in the present review, it was found that cannabis use during pregnancy has a multifaceted aggravating effect on the newborn and its subsequent development. In terms of physical health, the intrauterine exposure to cannabis is an important risk factor for the birth of a newborn with a lower weight^5,9,11,14,18^, premature for gestational age^5,9,13,14,17^, as well as for their hospitalization in an NICU^5,14,17^. As regards infancy and later infancy, it was found that at the age of 18 months, cannabis is an aggravating factor for the development of behavioral problems, as well as attention deficits, with more frequent occurrences of these in girls^12^. In contrast, the father’s habits have not been shown to affect the newborn so far^12^. An additional important finding is the effect of cannabis on DNA methylation^16^. The use of cannabis during pregnancy causes changes in human DNA positions, which affect the expression of characteristics.

With regard to the selection process of the study population as well as the information acquisition, a wide variety of methods were found which used different criteria. Several researchers used questionnaires^11-14,16-18^ as the main means of approach, which were basically answered during a routine visit to the physician. The main danger regarding the reliability of the results is related to the woman’s willingness to respond with sincerity. Many women who were invited to participate in this way probably did not respond honestly fearing negative criticism about their choice. Although cannabis use is now the subject of extensive study, with a wealth of scientific data constantly coming to light, the female population still feels quite guilty, given that cannabis use is not legal in many countries. In such cases, the fear of being targeted is intense, which may lead many women to refuse to participate in such studies. At the same time, when the study concerned the simultaneous use of cannabis and tobacco, it was not always easy to separate each effect.

In addition, when comparing studies, it has been found that the age range of participants varies from study to study, without a universal selection criterion. There are studies in which women under the age of 18 years were excluded^5,11,14^ from participating in the study, unlike other studies where the age limit of participation was 15 years^17^. Another factor that differentiates the choice of sample is the medical history of each woman. Women who were pregnant with a twin, suffered from diabetes mellitus, consumed large quantities of alcohol or drug addiction (except cannabis) could not participate in the research^6,7^. This heterogeneity often excludes large population groups, whose possible participation would have radically changed the already known data.

### Strengths and limitations

The strengths of this study are based on the satisfactory number of primary studies included in the review. Due to the years of their implementation which cover a wide period of time, they demonstrate the variability of some information, but at the same time, they show the stability of some findings. The support of the international bibliography has enabled access to a large amount of information. A limitation is that the studies differed concerning the population approached. Nevertheless, taking everything into account, they all have produced similar results. This indicates that the consequences of cannabis use were the same, regardless of their documentation method. Also, the fact that the studies were conducted in different countries increases the strength of the findings, thus demonstrating race and ethnicity as independent factors.

## CONCLUSIONS

It would be useful for future studies to focus on finding additional reliable methods of identifying users of illegal substances and defining objective and universal criteria for conducting the relevant investigations. At the same time, as far as the state is concerned, it is necessary to create support groups for addicts, with emphasis on pregnant women. In these structures, the presence of a psychologist is important and also the active participation of gynecologists and midwives to monitor these women and provide appropriate obstetric care, since it has been found that cannabis affects the developing fetus. To assist health professionals in this effort, the establishment of universal guidelines/algorithms for the more effective management of such cases is needed. The organization of workshops informing the general population about cannabis as well as other drugs use will help to consolidate the perception that drugs, apart from the transient pleasure they provide to the individual, can only have harmful effects in the long-term. As a result, the population, in which women also belong, will be informed from an early age and they will be prevented from developing such habits. Finally, countries where the use of cannabis is prohibited, apart for medical purposes, should consider establishing a new legislative framework which will not be exclusively punitive, but will help the pregnant woman to admit that she is a user in order to seek help from the competent bodies.

## Data Availability

All sources used are available to the general public for further information and evaluation purposes. An extended bibliography is given in the Supplementary file.
