# Supplementary material for: Cannabis use during pregnancy and its effect on the fetus, newborn and later childhood: A systematic review
Source: Eur J Midwifery. 2023 Aug 4;7:19. doi: 10.18332/ejm/168727 (PMC10401888; doi:10.18332/ejm/168727)
Supplement: Supplementary file 1 [file EJM-7-19-s1.pdf]

Supplementary Material

1. Abel EL, Bush R, Dintcheff BA, Ernst CA. Critical periods for marihuana-induced intrauterine growth retardation in the rat. Neurobehavioral Toxicology and Teratology 1981; 3 (3): 351-354. doi: does not exist. **Reason for rejection**: The study population is animals.

2. Abel EL. Effects of prenatal exposure to cannabinoids. Effects of prenatal exposure to cannabinoids 1985; 59: 20-35. doi: does not exist. **Reason for rejection**: The full text is not available.

3. Bao C, Bao S. Neonate Death Due to Marijuana Toxicity to the Liver and Adrenals. The American journal of case reports 2019; 20: 1874–1878. doi: 10.12659/AJCR.919545 **Reason for rejection**: This is a case report.

4. Bell GL, Lau K. Perinatal and neonatal issues of substance abuse. Pediatric Clinics of North America 1995; 42 (2): 261 – 281. doi: 10.1016/S0031-3955(16)38946-5 **Reason for rejection**: This is a review.

5. Benevenuto SG, Domenico MD, Martins MAG, et al. Recreational use of marijuana during pregnancy and negative gestational and fetal outcomes: An experimental study in mice. Toxicology 2017; 376: 94-101. doi: 10.1016/j.tox.2016.05.020 **Reason for rejection**: The study population is animals

6. Bergeria CL, Heil SH. Surveying Lactation Professionals Regarding Marijuana Use and Breastfeeding. Breastfeeding Medicine 2015; 10 (7): 377-380. doi: 10.1089/bfm.2015.0051 **Reason for rejection**: It doesn't answer the question of the study.

7. Berenson AB, WIlkinson GS, Lopez LA. Effects of prenatal care on neonates born to drug-using women. Substance Use and Misuse 1996; 31 (8): 1063 – 1076. doi: 10.3109/10826089609072288 **Reason for rejection**: The full text was not available.

8. Berra A, Kamo S, Mohnot S, et al. The Association between Antenatal Maternal Self-reported Substance Use, Maternal Characteristics, and Obstetrical Variables. Journal of Addiction Medicine 2019; 13 (6): 464 – 469. doi:10.1097/ADM.0000000000000521. **Reason for rejection**: It refers more generally to the use of substance use

9. Blandthorn J, Forster DA, Love Veronica. Neonatal and maternal outcomes following maternal use of buprenorphine or methadone during pregnancy: Findings of a retrospective audit. Women and Birth

2011; 24 (1): 32 – 39. doi: 10.1016/j.wombi.2010.07.00 **Reason for rejection**: Not related to the topic.

10. Brown HL, Britton KA, Mahaffey D, Brizendine E, Hiett AK, Turnquest MA. Methadone maintenance in pregnancy : A reappraisal. American Journal of Obstetrics and Gynecology 1998; 179 (2): 459 – 463. doi: 10.1016/S0002-9378(98)70379-5 **Reason for rejection**: Not related to the topic.

11. Carlier J, Huestis M, Zaami S, Pichini S, Busardò FP. Monitoring Perinatal Exposure to Cannabis and Synthetic Cannabinoids. Therapeutic drug monitoring 2020; 42 (2): 194-204. doi: 10.1097/FTD.0000000000000667 **Reason for rejection:** This is a review.

12. Coleman-Cowger VH, Oga E, Peters EN, Mark K. Prevalence and Associated Birth Outcomes of Co-Use of Cannabis and Tobacco Cigarettes during Pregnancy. Neurotoxicology and Teratology2018; 68: 84-90. doi: 10.1016/j.ntt.2018.06.001 **Reason for rejection**: It is not a primary study.

13. Coles CD, Platzman KA, Smith I, James ME, Falek A. Effects of cocaine and alcohol use in pregnancy on neonatal growth and neurobehavioral status. Neurotoxicology and Teratology 1992; 14 (1): 23 – 33. doi: 10.1016/0892-0362(92)90025-6 **Reason for rejection**: Not related to the topic.

14. Crome IB, Kumar MT. Epidemiology of drug and alcohol use in young women. Seminars in Fetal and Neonatal Medicine 2007; 12 (2): 98 – 105. doi: 10.1016/j.siny.2006.12.002 **Reason for rejection**: Not related to the topic.

15. Davis E, Lee T, Weber T J, Budgen S. Cannabis use in pregnancy and breastfeeding : The pharmacist's role. CPJRPC 2020; 153(2) pp 95-100. doi: 10.1177/1715163519893395 **Reason for rejection**: This is a review.

16. Debelak K, Morrone WR, O'Grady KE, Jones HE. Buprenorphine + naloxone in the treatment of opioid dependence during pregnancy - Initial patient care and outcome data. American Journal on Addictions 2013; 22 (3): 252 – 254. doi: 10.1111/j.1521-0391.2012.12005.x **Reason for rejection**: Not related to the topic.

17. Dong C, Chen J, Harrington A, Vinod Yaragudri K, Hegde M, Hegde L V. Cannabinoid exposure during pregnancy and its impact on immune function. Cellural and Molecular Life Sciences 2018; 76 (4): 729-743. doi : 10.1007/s00018-018-2955-0 **Reason for rejection**: This is a review

18. Emery RL, Gregory MP, Grace JL, Levine MD. Prevalence and correlates of a lifetime cannabis use disorder among pregnant former tobacco smokers. Addictive behaviors 2015; 54: 52-58. doi:10.1016/j.addbeh.2015.12.008 **Reason for rejection**: It doesn't answer the question of the study.

19. Eyler FD, Behnke M, Conlon M, Woods NS, Wobie K. Birth outcome from a prospective, matched study of prenatal crack/cocaine use: II. Interactive and dose effects on neurobehavioral assessment. Pediatrics 1998; 101 (2): 237 – 241. doi : 10.1542/peds.101.2.237 **Reason for rejection**: Not related to the topic

20. Fischer G, Johnson RE, Eder H, et al. Treatment of opioid-dependent pregnant women with buprenorphine. Addiction 2000; 95 (2): 239 – 244. doi:10.1046/j.1360-0443.2000.95223910.x **Reason for rejection**: Not related to the topic.

21. Fonseca BM, Correia-da-Silva G, Almada M, Costa MA, Teixeira NA. The Endocannabinoid System in the Postimplantation Period: A Role during Decidualization and Placentation. International journal of endocrinology 2013. doi: 10.1155/2013/510540. **Reason for rejection**: It doesn't answer the question of the study.

22. Frank DA, Jacobs RR, Beeghly M, et al. Level of Prenatal Cocaine Exposure and Scores on the Bayley Scales of Infant Development: Modifying Effects of Caregiver, Early Intervention , and Birth Weight. Pediatrics 2002; 110(6): 1143-52. doi : 10.1542/peds.110.6.1143 **Reason for rejection**: Not related to the topic.

23. Frau R, Miczán V, Traccis F. Prenatal THC exposure produces a hyperdopaminergic phenotype rescued by pregnenolone. Nature neuroscience 2019; (12): 1975- 1985. doi : 10.1038/s41593-019-0512-2 **Reason for rejection**: It refers to animals and full text is not available.

24. Geraldine GA, Aiton NR. Intrauterine illicit drug exposure and neurodevelopmental outcomes for children: how current literature informs management and assessment. Paediatrics and Child Health (United Kingdom) 2017; 27 (8): 383 – 387. doi: 10.1016/j.paed.2017.05.003 **Reason for rejection**: This is a review and it doesn't answer the question of the study

25. Gibson GT, Baghurst PA, Colley DP. Maternal Alcohol, Tobacco and Cannabis Consumption and the Outcome of Pregnancy. Australian and New Zealand Journal of Obstetrics and Gynaecology 1983; 23 (1): 15 – 19. doi:10.1111/j.1479-828X.1983.tb00151.x **Reason for rejection**: It is not a primary study.

26. Gobbi G, Atkin T, Zytynski T, et al. Association of Cannabis Use in Adolescence and Risk of Depression, Anxiety, and Suicidality in Young Adulthood : A Systematic Review and Meta-analysis. JAMA Psychiatry 2019; (4): E1-E9. doi: 10.1001/jamapsychiatry.2018.4500 **Reason for rejection**: This is a systematic review and meta-analysis.

27. Godleski SA, Shisler S, Eiden RD, Huestis MA. Co-use of tobacco and marijuana during pregnancy: Pathways to externalizing behavior problems in early childhood. Neurotoxicology and Teratology 2018; 69: 39-48. doi : 10.1016/j.ntt.2018.07.003 Reason for rejection: It doesn't answer the question of the study.

28. Goel N , Beasley D, Rajkumar V, Banerjee S. Perinatal outcome of illicit substance use in pregnancy-comparative and contemporary socio-clinical profile in the UK. European Journal of Pediatrics 2011; 170 (2): 199 – 205. doi: 10.1007/s00431-010-1284-6 **Reason for rejection**: It doesn't answer the question of the study.

29. Goldschmidt L, Richardson GA, Larkby C, Day NL. Early marijuana initiation: The link between prenatal marijuana exposure, early childhood behavior, and negative adult roles. Neurotoxicology and Teratology 2016; 58: 40-45. doi: 10.1016/j.ntt.2016.05.011 **Reason for rejection** : The results of the study do not answer the question of the study.

30. Goldschmidt L, Richardson GA, Willford JA, Severtson SG, Day NL (2011). School achievement in 14-year-old youths prenatally exposed to marijuana. Neurotoxicology and Teratology 2011; 34 (1): 161-167. doi: 10.1016/j.ntt.2011.08.009 **Reason for rejection**: It refers to teenagers.

31. Gopalan P, Moses-Kolko E, Valpey R, Shenai N, Smith E. Benzodiazepine withdrawal in pregnant women with opioid use disorders: An observational study of current clinical practices at a tertiary obstetrical hospital. General Hospital Psychiatry 2019; 57: 29-33. doi: 10.1016/j.genhosppsych.2018.12.005 **Reason for rejection**: It is not related to the topic.

32. Gnofam M, Allshouse A, Stickrath EH, Metz TD. Impact of marijuana legalization on prevalence of maternal marijuana use and perinatal outcomes. American Journal of Perinatology 2020; 37 (1): 59 – 65. doi:10.1055/s-0039-1696719 **Reason for rejection**: It doesn't answer the question of the study and the full text is not available.

33. Grant SK, Petroff R, Isoherranen N, Stella N, Burbacher MT. Cannabis Use during Pregnancy :Pharmacokinetics and Effects on Child Development . Pharmacology & therapeutics 2017; 182: 133-

151. doi: 10.1016/j.pharmthera.2017.08.014 **Reason for rejection**: This is a review.

34. Gunn, JKL, Rosales BC , Center KE, et al. Prenatal exposure to cannabis and maternal and child health outcomes: a systematic review and meta-analysis. BMJ Open 2016; (4): 1-8. doi : 10.1136/bmjopen-2015-009986 **Reason for rejection**: This is a systematic review and meta-analysis.

35. Hill M, Reed K. Pregnancy, breast-feeding, and marijuana: A review article. Obstetrical and Gynecological Survey 2013; 68 (10): 710 – 718. doi:10.1097/01.ogx.0000435371.51584.d1 **Reason for rejection**: This is a review.

36. Hingson R, Zuckerman B, Amaro H, et al. Maternal Marijuana Use and Neonatal Outcome: Uncertainty Posed by Self-Reports. American Journal of Public Health 1986; (6): 667-669. doi: 10.2105/ajph.76.6.667 **Reason for rejection**: It doesn’t answer the question of the study.

37. Huestis MA, Choo RE. Drug abuse's smallest victims: In utero drug exposure. Forensic Science International 2002; 128 (1-2): 20 – 30. doi:10.1016/S0379-0738(02)00160-3 **Reason for rejection** : This is a conference paper and it doesn’t answer the question of the study.

38. Huizink AC. Prenatal cannabis exposure and infant outcomes: Overview of studies . Progress in Neuro-Psychopharmacology and Biological Psychiatry 2014; 52: 45 – 52. doi: 10.1016/j.pnpbp.2013.09.014 **Reason for rejection**: This is an overview of already existing studies.

39. Jansson LM, Velez ML. Infants of drug-dependent mothers. Pediatrics in Review 2011; 32 (1): 5 – 13. doi:10.1542/pir.32-1-5 **Reason for rejection**: It doesn’t answer the question of the study.

40. Johnson S, Martin PR. Transitioning from methadone to buprenorphine maintenance in management of opioid use disorder during pregnancy. American Journal of Drug and Alcohol Abuse 2018; 44 (3): 310 – 316. doi:10.1080/00952990.2017.1363218 **Reason for rejection**: It’s not related to the topic

41. Keegan J, Parva M, Finnegan M, Gerson A, Belden M. Addiction in pregnancy. Journal of Addictive Diseases 2010; 29 (2): 175 – 191. doi: 10.1080/10550881003684723 **Reason for rejection**: This is a review and doesn’t answer the question of the study.

42. Logan BA, Brown MS., Hayes MJ. Neonatal abstinence syndrome: Treatment and pediatric outcomes .Clinical Obstetrics and

Gynecology 2013; 56 (1): 186 – 192. doi:10.1097/GRF.0b013e31827feea4 **Reason for rejection**: This is a conference paper and doesn't answer the question of the study.

43. Louw KA. Substance use in pregnancy: The medical challenge. Obstetric Medicine 2018; 11 (2) :54-66. doi: 10.1177/1753495X17750299 Reason for rejection: This is a review.

44. Mark K, Pierce E., Joseph D, Crimmins S. Interaction with the justice system and other factors associated with pregnant women's self-report and continuation of use of marijuana. Drug and Alcohol Dependence 2020; 206: 1-5. doi:10.1016/j.ntt.2016.05.011 **Reason for rejection**: It doesn't answer the question of the study.

45. Mcallister-Williams RH, Baldwin DS, Cantwell R, et al. British Association for Psychopharmacology consensus guidance on the use of psychotropic medication preconception, in pregnancy and postpartum. Journal of Psychopharmacology 2017; 31 (5): 519 – 552. doi: 10.1177/0269881117699361 **Reason for rejection**: It's not related to the topic.

46. McCauley-Elsom K, Kulkarni J. Managing psychosis in pregnancy. Australian and New Zealand Journal of Psychiatry 2007; 41 (3): 289 – 292. doi:10.1080/00048670601172798 **Reason for rejection**: It's not related to the topic.

47. Mertz TD, Allshouse AA, Hogue CJ, et al. Maternal marijuana use, adverse pregnancy outcomes, and neonatal morbidity. American 53 Journal of Obstetrics and Gynecology 2017; 217 (4): 478.e1- 478.e.7. doi: 10.1016/j.ajog.2017.05.050 **Reason for rejection**: This is a secondary analysis.

48. Mertz TD, Stickrath EH. Marijuana use in pregnancy and lactation: a review of the evidence. American Journal of Obstetrics and Gynecology 2015; 213 (6):761-778. doi: 10.1016/j.ajog.2015.05.025 **Reason for rejection**: This is a review.

49. Metz TD, Borgelt LM. Marijuana Use in Pregnancy and While Breastfeeding. Obstetric Gynecology 2018; 132 (5): 1198-1210. doi: 10.1097/AOG.0000000000002878 **Reason for rejection**: This is not a primary study.

50. Minnes S, Lang A, Singer L. Prenatal Tobacco, Marijuana, Stimulant, and Opiate Exposure: Outcomes and Practice Implications. National Institute on Drug Abuse 2011; (1): 57-70. doi: 10.1097/AOG.0000000000002878 **Reason for rejection**: This is a review.

51. Monnier D, Tucciarone D, Fair S. Multidisciplinary Management Pulmonary Arterial Hypertension in Pregnancy. JOGNN - Journal of Obstetric, Gynecologic, and Neonatal Nursing 2015; 44: S84. doi:10.1111/1552-6909.12593 **Reason for rejection**: It's not related to the topic.

52. Morris CV, DiNieri JA, Szutorisz H, Hurd YL. Molecular mechanisms of maternal cannabis and cigarette use on human neurodevelopment. The European journal of neuroscience 2011;(10): 1574-1583. doi: 10.1111/j 1460-9568.2011.07884.x **Reason for rejection**: This is a review.

53. Passey ME, Sanson-Fisher RW, D'Este CA, Stirling JM. Tobacco, alcohol and cannabis use during pregnancy: Clustering of risks. Drug and Alcohol Dependence 2014; 134: 44-50. doi: 10.1016/j.drugalcdep.2013.09.008 **Reason for rejection**: It doesn't answer the question of the study.

54. Petrangelo A, Czuzoj-Shulman N, Balayla J, Abenhaim HA. Cannabis Abuse or Dependence During Pregnancy: A Population-Based Cohort Study on 12 Million Births. Journal of Obstetrics and Gynaecology Canada 2019; 41 (5): 623 – 630 doi:10.1016/j.jogc.2018.09.009 **Reason for rejection**: The full text is not available

55. Rebbe R, Mienko JA ,Brown E , Rahbar-Rowhani A. Hospital Variation in Child Protection Reports of Substance Exposed Infants. The Journal of Pediatrics 2019; 208: 141-147E2. doi:10.1016/j.jpeds.2018.12.065 **Reason for rejection** : It doesn't answer the question of the study.

56. Richardson GA, Hamel SC, Goldschmidt L, Day NL. The effects of prenatal cocaine use on neonatal neurobehavioral status. Neurotoxicology and Teratology 1996; 18 (5): 519 – 528 doi:10.1016/0892-0362(96)00062-1 **Reason for rejection**: It is not related to the topic.

57. Roncero C, Valriberas-Herrero I, Mezzatesta-Gava M, Villegas L J, Aguilar L, Grau-López L. Cannabis use during pregnancy and its relationship with fetal developmental outcomes and psychiatric disorders. A systematic review. Reproductive health 2020; 17 (25): 1-9. doi: 10.1186/s12978-020-0880-9. **Reason for rejection**: This is a systematic review.

58. Roth CK, Satran LA, Smith SM(2015). Marijuana Use in Pregnancy. Nursing for Women's Health 2015; 19 (5): 431 – 437. doi:10.1111/1751-486X.12235 **Reason for rejection**: This is not a primary study and the full text is not available

59. Ryan SA, Ammerman SD, O' Connor ME. Marijuana use During pregnancy And Breastfeeding: Implications for Neonatal and Childhood Outcomes. Pediatrics 2018; 142 (3): 1-11. doi: 10.1542/peds.2018-1889 **Reason for rejection**: This is not a primary study

60. Sharapova SR, Phillips E, Sirocco K , Kaminski JW, Leeb RT, Rolle I. Effects of Prenatal Marijuana Exposure on Neuropsychological Outcomes in Children Aged 1-11 Years: A Systematic Review. Paediatric and perinatal epidemiology 2018; 32 (6): 512-532. doi: 10.1111/ppe. 12505 **Reason for rejection**: This is a systematic review.

61. Shi Y, Zhong S. Trends in Cannabis Use Disorder among Pregnant Women in the U.S., 1993–2014. Journal of general internal medicine 2017; 33 (3): 245-246. doi: 10.1007/s11606-017-4201-0 **Reason for rejection**: It doesn't answer the question of the study.

62. Shieh C, Kravitz M. Severity of drug use, initiation of prenatal care, and maternal-fetal attachment in pregnant marijuana and cocaine/heroin users JOGNN - Journal of Obstetric, Gynecologic, and Neonatal Nursing 2006; 35 (4): 499 – 508. doi:10.1111/j.1552-6909.2006.00063.x **Reason for rejection**: It doesn't answer the question of the study.

63. Singer LT, Moore DG, Fulton S, et al. Neurobehavioral outcomes of infants exposed to MDMA (Ecstasy) and other recreational drugs during pregnancy. Neurotoxicology and Teratology 2012; 34 (3): 303 – 310. doi:10.1016/j.ntt.2012.02.001 **Reason for rejection**: It is not related to the topic.

64. Singer LT, Yamashita T, Hawkins S, Cairns D, Baley J, Kliegman R. Increased incidence of intraventricular hemorrhage and developmental delay in cocaine-exposed, very low birth weight infants. The Journal of Pediatrics 1994; 124 (5 PART 1): 765 – 771. doi:10.1016/S0022-3476(05)81372-1 **Reason for rejection**: It is not related to the topic.

65. Skelton KR, Hecht AA, Benjamin-Neelon SE. Recreational cannabis legalization in the US and maternal use during the preconception, prenatal and postpartum periods. International Journal of Environmental Research and Public Health 2020; 17 (3): 1-11. doi: 10.3390/ijerph17030909 **Reason for rejection**: It doesn't answer the question of the study.

66. Smith LM, LaGasse LL, Derauf C, et al. Prenatal methamphetamine use and neonatal neurobehavioral outcome. Neurotoxicology and Teratology 2008; 30 (1): 20-28. doi: 10.1016/j.ntt.2007.09.005 **Reason for rejection**: It is not related to the topic.

67. Sonon Ke, Richardson GA , Cornelius JR, Kim KH , Day NL. Prenatal Marijuana Exposure Predicts Marijuana Use in Young Adulthood. Neurotoxicology and Teratology 2014; 47: 10-15. doi: 10.1016/j.ntt.2014.11.003 **Reason for rejection**: It doesn't answer the question of the study.

68. Stone KC, LaGasse LL, Lester BM, et al. The Maternal Lifestyle Study: Sleep Problems in Children with Prenatal Substance Exposure. The Maternal Lifestyle Study. Archives of Pediatrics and Adolescent Medicine 2010; 164 (5): 452-456 doi: 10.1001/archpediatrics.2010.52 **Reason for rejection**: It doesn't answer the question of the study.

69. Sun X, Dey SK. Endocannabinoid Signaling in Female Reproduction. ACS CHEMICAL NEUROSCIENCE 2012; 3 (5): 349-351 doi: 10.1021/cn300014e **Reason for rejection** : This is a conference paper and doesn't answer the question of the study.

70. Thompson R, De Jong K, Lo J. Marijuana Use in Pregnancy: A Review. Obstetrical & gynecological survey 2019; 74 (7): 415–428. doi: 10.1097/OGX.0000000000000685 **Reason for rejection**: This is a review

71. Tirado-Muñoz J, Lopez-Rodriguez AB, Fonseca F, Farré M, Torrens M, Viveros MP. Effects of cannabis exposure in the prenatal and adolescent periods: Preclinical and clinical studies in both sexes. Frontiers in Neuroendocrinology 2020; 57: 1-22. doi: 10.1016/j.yfrne.2020.100841 **Reason for rejection**: This is a review.

72. Traccis F, Frau R, Melis M. Gender Differences in the Outcome of Offspring Prenatally Exposed to Drugs of Abuse. Frontiers in Behavioral Neuroscience 2020; 145 (72). doi:10.3389/fnbeh.2020.00072 **Reason for rejection**: This is a review not related to the topic

73. Viteri OA. Mendez-Figueroa H, Pedroza C, Leon MG, Sibai BM, Chauhan SP. Relationship between Self-Reported Maternal Substance Abuse and Adverse Outcomes in the Premature Newborn. American Journal of Perinatology 2016. 33 (2): 165 – 171. doi: 10.1055/s-0035-1563549 **Reason for rejection**: This is a conference paper.

74. Von Mandach U. Drug use in pregnancy[Drogen in der schwangerschaft]. Therapeutische Umschau 2005; 62 (1): 29 – 35.

doi:10.1024/0040-5930.62.1.29 **Reason for rejection**: This is a review not related to the topic.

75. Welffens K, Derisbourg S, Costa E, et al. The “Cocoon,” first alongside midwifery-led unit within a Belgian hospital: Comparison of the maternal and neonatal outcomes with the standard obstetric unit over 2 years. Birth 2020; 47(1): 115 – 122. doi: 10.1111/birt.12466 **Reason for rejection**: It is not related to the topic

76. Wong SPW, Twynstra J, Gilliland JA, Cook JL, Seabrook J. Risk Factors and Birth Outcomes Associated with Teenage Pregnancy: A Canadian Sample. Journal of Pediatric and Adolescent Gynecology 2020; 33 (2): 153-159. doi: 10.1016/j.jpag.2019.10.006 **Reason for rejection:** It doesn’t answer the question of the study.

77. Young-Wolf KC, Sarovar V, Tucker LY, Conway A, Alexeeff S, Weisner C, Armstrong MA, Goler N. Self-reported Daily,Weekly, and Monthly Cannabis Use Among Women Before and During Pregnancy. JAMA Network open 2019A; 2 (7): 1-10. doi: 10.1001/jamanetworkopen.2019.6471 **Reason for rejection**: It doesn’t answer the question of the study.

78. Young-Wolff KC, Sarovar V, Tucker LY, et al. Trends in marijuana use among pregnant women with and without nausea and vomiting in pregnancy, 2009 to 2016. Drug and Alcohol Dependence 2019B; 196: 66-70. doi: 10.1016/j.drugalcdep.2018.12.009 **Reason for rejection**: It doesn’t answer the question of the study.

© 2023 Ainiti D. F. et al.
